# Intrathecal pemetrexed in NSCLC patients with leptomeningeal metastasis

**DOI:** 10.3332/ecancer.2024.1792

**Published:** 2024-10-31

**Authors:** Vanita Noronha, Vijay Patil, Zoya Peelay, Monica Reddy Yallala, Nandini Menon, Minit Shah, Shatabdi Chakraborty, Kumar Prabhash

**Affiliations:** Department of Medical Oncology, Tata Memorial Hospital, Homi Bhabha National Institute, Mumbai 400012, India

**Keywords:** non-small cell lung cancer, leptomeningeal metastasis, osimertinib, pemetrexed, intrathecal chemotherapy

## Abstract

Spread of lung cancer to the leptomeninges is rare and difficult to treat. Standard therapy comprises CNS-penetrant targeted agents with or without intrathecal chemotherapy. We performed a retrospective analysis of 16 patients with advanced NSCLC and leptomeningeal disease treated with intrathecal pemetrexed 50 mg. All tumours were adenocarcinoma histology; 13 (81.3%) had EGFR mutations, and 3 (18.8%) had no targetable mutations. Prior therapies included EGFR-directed tyrosine kinase inhibitors (TKI) with/without chemotherapy/antiangiogenic agents (9 [56.3%]), chemotherapy alone (4 [25%]), intrathecal methotrexate with/without hydrocortisone (3 [18.9%]), and radiation (12 [75%]). Presenting symptoms of leptomeningeal disease included headache (10 [62.5%]), dizziness (8 [50%]), and seizures (7 [43.8%]).

Systemic therapy administered along with intrathecal pemetrexed included osimertinib (5 [31.3%]), gefitinib in 1 (6.3%), chemotherapy in 4 (25%) (pemetrexed + carboplatin-2, cisplatin + etoposide-1, paclitaxel-1), chemotherapy + oral TKI in 5 (31.3%) and no systemic therapy in 1 (6.3%). Neurological symptoms following intrathecal pemetrexed included headache in 1 (6.3%) patient which was likely due to raised intracranial pressure from underlying leptomeningeal disease, and anxiety/uneasiness in 1 (6.3%). Grade 3 or higher toxicities included thrombocytopenia (6 [37.5%]), anaemia (4 [25%]), neutropenia (4 [25%]), febrile neutropenia (3 [18.8%]), mucositis (4 [25%]), diarrhoea (1 [6.3%]), rash (1 [6.3%]) and hypokalemia (1 [6.3%]. Most toxicities were likely caused by systemic chemotherapy, rather than by intrathecal pemetrexed. Intrathecal pemetrexed was delayed in 9 (56.3%) patients, due to cytopenias/febrile neutropenia (8 [50%]) and poor general condition (1 [6.3%]). Median OS from diagnosis of leptomeningeal disease was 7.5 months (95% CI: 1.2–13.8). Median OS from start of intrathecal pemetrexed was 2.7 months (95% CI, 1.1–4.3).

Thus, intrathecal pemetrexed combined with systemic antitumor therapy was tolerable, with promising clinical outcomes in patients with NSCLC and leptomeningeal disease. It is important to explore this option, especially in driver mutation-negative NSCLC patients.

## Efficacy and safety of intrathecal pemetrexed for leptomeningeal metastases of non-small cell lung cancer

Non-small cell lung cancers (NSCLCs) can present with leptomeningeal metastasis (LM) as a rare complication but with high mortality [1]. Treatment options for this condition may include surgery, radiation therapy, systemic or intrathecal chemotherapy, molecularly targeted agents and immunotherapy [2, 3]. There are no standard guidelines for the management of LM; the common approach is intrathecal chemotherapy [4]. Fan *et al* [5] had reported that intrathecal pemetrexed at 50 mg in *EGFR*-mutant NSCLC with LM was promising, with an 84.6% clinical response rate and a median overall survival (OS) of 9 months (95% CI, 6.6–11.4) [5]. Hong *et al* [6] have also described the use of intrathecal pemetrexed.

We conducted an institutional review board-approved retrospective study of patients with NSCLC and LM who were treated with intrathecal pemetrexed. The objective was to examine the clinical characteristics of NSCLC patients with LM and evaluate the tolerability of intrathecal pemetrexed in combination with systemic treatment. We collected information on 16 NSCLC patients who had received intrathecal pemetrexed treatment for LM, planned in a multidisciplinary clinic. Patients were identified based on positive cerebrospinal fluid cytology (*n* = 15 [93.8%]) or characteristic imaging features, such as enhancement of the leptomeninges or widening of the ventricles (*n* = 12 [75%]). Toxicity was scored as per the common terminology criteria for adverse events, version 5. OS was estimated using the Kaplan–Meier method.

Of the 16 patients, 10 (62.5%) were men. Performance status was 1 in 4 (25%) patients, 2 in 4 (25%) and 3 in 8 (50%). All tumours were of adenocarcinoma histology. On molecular testing, 13 (81.3%) had *EGFR* mutation (exon 19 deletion-7 [43.8%], exon 21 L858R mutation-4 [25%], exon 20 S768I-1 [6.3%], exon 20 insertion-1 [6.3%]), 1 (6.3%) had TP53 mutation and 2 (12.5%) had no driver mutations. There were 2 (12.5%) patients with *EGFR* sensitising mutations who had acquired *EGFR* T790M resistance mutations at progression. Before the LM diagnosis, therapies received included EGFR tyrosine kinase inhibitors (TKI) (+/- chemotherapy/antiangiogenic agents) in 9 (56.3%), chemotherapy alone in 4 (25%), intrathecal methotrexate +/- hydrocortisone in 3 (18.9%) and radiation in 12 (75%). Common presenting symptoms included headache in ten patients (62.5%), dizziness in 8 (50%) and seizures in 7 (43.8%). Intrathecal pemetrexed was dosed at 50 mg and delivered through a lumbar puncture in all patients. Systemic therapy administered along with intrathecal pemetrexed included osimertinib in 5 (31.3%) patients, gefitinib in 1 (6.3%), chemotherapy in 4 (25%) (pemetrexed + carboplatin-2, cisplatin + etoposide-1, paclitaxel-1) chemotherapy + oral TKI in 5 (31.3%) and no systemic therapy in 1 (6.3%). Neurological symptoms following intrathecal pemetrexed included headache in 1 (6.3%) patient which was likely due to continuing raised intracranial pressure from the leptomeningeal disease, and anxiety/uneasiness in 1 (6.3%) patient. Grade 3 or higher toxicities (many of which were attributable to the systemic therapies administered, rather than the intrathecal pemetrexed) included thrombocytopenia (6 [37.5%]), anaemia (4 [25%]), neutropenia (4 [25%]), febrile neutropenia (3 [18.8%]), mucositis (4 [25%]), diarrhoea (1 [6.3%]), rash (1 [6.3%]) and hypokalemia (1 [6.3%]. Adverse events were managed with supportive care, transfusion for low counts in 2 (12.5%) and GCSF in 1 (6.3%). Administration of intrathecal pemetrexed was delayed in 9 (56.3%) patients, due to cytopenias/febrile neutropenia in 8 (50%) and poor general condition in 1 (6.3%). The median OS after diagnosis of LM was 7.5 months (95% CI: 1.2–13.8). The median OS from the start of intrathecal pemetrexed was 2.7 months (95% CI, 1.1–4.3).

Thus, intrathecal pemetrexed combined with systemic antitumor therapy was tolerable, with promising clinical outcomes in NSCLC patients with LM. It is important to explore this option, especially in driver mutation-negative NSCLC patients.

## Conflicts of interest

None.

## Funding

The study was self-funded. We did not receive any funding for this study.

## Data availability statement

The authors confirm that the data supporting the findings of this study are available from the corresponding author Dr. Kumar Prabhash on request.
